# High Ambient Temperature Regulated the Plant Systemic Response to the Beneficial Endophytic Fungus *Serendipita indica*

**DOI:** 10.3389/fpls.2022.844572

**Published:** 2022-03-16

**Authors:** Xiao-Jie Chen, Yue-Qing Yin, Xin-Meng Zhu, Xue Xia, Jia-Jia Han

**Affiliations:** ^1^Yunnan Key Laboratory of Plant Reproductive Adaptation and Evolutionary Ecology, Institute of Biodiversity, School of Ecology and Environmental Science, Yunnan University, Kunming, China; ^2^Laboratory of Ecology and Evolutionary Biology, State Key Laboratory for Conservation and Utilization of Bio-Resources in Yunnan, Yunnan University, Kunming, China

**Keywords:** plant systemic response, PIF4, *Serendipita indica*, high ambient temperature, endophytic fungus

## Abstract

Most plants in nature establish symbiotic associations with endophytic fungi in soil. Beneficial endophytic fungi induce a systemic response in the aboveground parts of the host plant, thus promoting the growth and fitness of host plants. Meanwhile, temperature elevation from climate change widely affects global plant biodiversity as well as crop quality and yield. Over the past decades, great progresses have been made in the response of plants to high ambient temperature and to symbiosis with endophytic fungi. However, little is known about their synergistic effect on host plants. The endophytic fungus *Serendipita indica* colonizes the roots of a wide range of plants, including Arabidopsis. Based on the Arabidopsis-*S. indica* symbiosis experimental system, we analyzed the synergistic effect of high ambient temperature and endophytic fungal symbiosis on host plants. By transcriptome analysis, we found that DNA replication-related genes were significantly upregulated during the systemic response of Arabidopsis aboveground parts to *S. indica* colonization. Plant hormones, such as jasmonic acid (JA) and ethylene (ET), play important roles in plant growth and systemic responses. We found that high ambient temperature repressed the JA and ET signaling pathways of Arabidopsis aboveground parts during the systemic response to *S. indica* colonization in roots. Meanwhile, PIF4 is the central hub transcription factor controlling plant thermosensory growth under high ambient temperature in Arabidopsis. PIF4 is also involving JA and/or ET signaling pathway. We found that PIF4 target genes overlapped with many differentially expressed genes (DEGs) during the systemic response, and further showed that the growth promotion efficiency of *S. indica* on the *pif4* mutant was higher than that on the wild-type plants. In short, our data showed that high ambient temperature strengthened the growth promotion effect of *S. indica* fungi on the aboveground parts of the host plant Arabidopsis, and the growth promotion effect of the systemic response under high ambient temperature was regulated by PIF4.

## Introduction

Beneficial endophytic fungi in soil establish symbiotic interactions with most plants in natural ecosystems and induced a systemic response in the aboveground parts (stems, leaves, etc.) of host plants (Pieterse et al., 2014; Weiss et al., 2016). Beneficial endophytic fungi can improve host plants’ resistance to biotic and abiotic stresses, and promote the growth and fitness of host plants (Cameron et al., 2013; Pieterse et al., 2014; van der Heijden et al., 2015). *Serendipita indica* (syn. *Piriformospora indica*) is a *Sebacinales* fungus first isolated from the rhizosphere in deserts and symbiotically colonizes the roots of many plants, including Brassicaceae plants (such as the model plant *Arabidopsis thaliana*), which are known as non-host plants for ectomycorrhizae and arbuscular mycorrhizae (Verma et al., 1998; Peskan-Berghofer et al., 2004; Zuccaro et al., 2011; Qiang et al., 2012; Weiss et al., 2016). *S. indica* colonization in roots has a significant growth promotion effect on the biomass of the aboveground parts of host plants, including Arabidopsis (Peskan-Berghofer et al., 2004; Waller et al., 2005; Shahollari et al., 2007; Abdelaziz et al., 2019; Li et al., 2021). Previous studies in root endophytism and/or mycorrhizal association have mainly focused on the symbiosis signaling pathways that are necessary for the establishment of successful symbiotic relationships in plant roots (Lahrmann et al., 2015; Zipfel and Oldroyd, 2017; Rey and Jacquet, 2018), while the systemic responses of the aboveground parts of host plants are largely unknown.

For symbiotic interactions with Arabidopsis, *S. indica* endophytically colonizes root epidermal and cortex cells mainly in the meristematic zone and does not penetrate the central cylinder (Jacobs et al., 2011). The growth promotion effect of *S. indica* is due to the regulation of nutrients (phosphate, nitrate, etc.) uptake (Sherameti et al., 2005; Yadav et al., 2010; Bakshi et al., 2017; Prasad et al., 2019) and hormone [auxin, jasmonic acid (JA), salicylic acid (SA), etc.] homeostasis or signal transduction in host plants (Vadassery et al., 2008; Camehl et al., 2010; Jacobs et al., 2011; Hilbert et al., 2012; Lahrmann et al., 2015; Peskan-Berghofer et al., 2015). *S. indica* also enhances the resistance of host plants to biotic stress (Waller et al., 2005; Sun et al., 2014: Ye et al., 2019; Li et al., 2021) and abiotic stress conditions, such as drought (Sherameti et al., 2008), salt stress (Jogawat et al., 2013, 2016; Abdelaziz et al., 2017, 2019; Lanza et al., 2019; Luo et al., 2021) and cold stress (Jiang et al., 2020, 2021). Thus, Arabidopsis-*S. indica* symbiosis represents a model system in studying host plant–beneficial microbe interactions at the molecular level (Peskan-Berghofer et al., 2004). In the context of global warming, temperature elevation has become a major environmental factor affecting plant growth and development. However, it is unclear how high ambient temperature affects plant growth and development in the context of fungal symbiosis.

Exposure to high ambient temperature results in plant development changes termed thermomorphogenesis, which is characterized by hypocotyl elongation and petiole elongation in Arabidopsis (Koini et al., 2009; Quint et al., 2016). In Arabidopsis, high ambient temperature (greater than 22°C, approximately 28°C) is perceived by several thermosensors (Chen et al., 2021; Zhang et al., 2021). In response to high ambient temperature, phytochrome B (phyB) and/or EARLY FLOWERING3 (ELF3), which are major thermosensors in Arabidopsis, relieved their inhibitory effects on the temperature signal transduction component PHYTOCHROME INTERCTING FACTOR 4 (PIF4) (Jung et al., 2016, 2020; Legris et al., 2016; Zhang et al., 2021). PIF4 is the central hub transcription factor controlling thermosensory growth and development in Arabidopsis (Zhang et al., 2021). When Arabidopsis plants shift to high ambient temperature, PIF4 binds to the promoter regions of downstream thermoresponsive target genes and upregulates their expression. These genes are mainly involved in cell elongation, as well as plant hormone biosynthesis and response (Sun et al., 2012; Nieto et al., 2015; Gangappa and Kumar, 2017; Zhang et al., 2021). Meanwhile, PIF4 negatively regulates plant immunity and coordinates thermosensory growth and immunity (Gangappa et al., 2017). PIF4 also regulates JA and ethylene (ET) signaling pathways (Yamashino et al., 2013; Zhang et al., 2018; Xiang et al., 2020). Plant hormones (JA, ET, etc.) play an important role in plant systemic responses (Pieterse et al., 2014), but it is unclear whether PIF4 is involved.

In this study, we performed the cocultivation of *S. indica* with Arabidopsis under different temperatures (22, 25, and 28°C) and found that high ambient temperature enhanced the growth promotion effect of *S. indica* on Arabidopsis aboveground parts. By transcriptome analysis of the systemic response of the host plant Arabidopsis, we comprehensively analyzed the differentially expressed genes (DEGs) between fungal symbiotic and non-symbiotic plants at normal (22°C) and high ambient temperatures (25 and 28°C). We found that DNA replication-related genes were significantly upregulated during the systemic response of Arabidopsis aboveground parts to *S. indica* colonization. High ambient temperatures decreased the number of upregulated DEGs involving the JA and/or ET pathways, which implied repression of the JA and/or ET signaling pathways during the systemic response. Meanwhile, we also found that many DEGs overlapped with PIF4 target genes, and the growth promotion efficiency of *S. indica* on the *pif4* mutant was higher than that on wild-type (WT) plants at warm temperatures (28°C).

## Materials and Methods

### Co-cultivation of Arabidopsis Plant and *Serendipita indica* Fungus

Wild-type plant is *A. thaliana* Col-0 ecotype, and Arabidopsis *pif4* loss-of-function mutant is *pif4-101* (Wang et al., 2018). Arabidopsis was grown on half-strength MS medium with 1.2% sucrose (1/2 MS). The seeds were stratified at 4°C for 3 days and then first grown at 22°C under short-day growth condition (12:12 h, day:night photoperiod, 7000 LX light) in growth chambers. *Serendipita indica* (syn. *P. indica*, DSM11827) fungus were grown and conserved in PDA medium (200 g L^–1^ potato, 20 g L^–1^ dextrose, and 20 g L^–1^ agar). For cocultivation with *S. indica*, 9-day-old Arabidopsis seedlings were inoculated with *S. indica* in PNM medium plates (Johnson et al., 2011). *S. indica*-incubated (Si) and control (CK) samples were grown at 22, 25, or 28°C under short-day growth condition. The statistics of fresh weight were performed on 7 and 14 days post inoculation (dpi). Fresh weights of each seedling were the average weight of at least ten seedlings, which grew in the same plate. The mean and standard error statistics of fresh weights included at least three biological replicates for each sample. Significance analysis of differences were performed by *t*-test.

### Transcriptome and Reverse Transcription-Quantitative PCR Analysis

The aboveground parts of *S. indica*-inoculated Arabidopsis at 7 dpi were harvested for transcriptome and reverse transcription-quantitative PCR (RT-qPCR) analysis. Total RNA extracted from Si and CK samples grown at 22, 25, or 28°C was used for sequencing with the NovaSeq 6000 sequencer by Majorbio (Shanghai, China). The clean reads were mapped to the *A. thaliana* TAIR10 database. Bioinformatics analyses of the transcriptome were performed using the online platform of Majorbio Cloud Platform^1^ and jvenn Venn diagram viewer (Bardou et al., 2014). Briefly, to identify DEGs between different samples, the expression level of each transcript was calculated according to the transcripts per million reads (TPM) method. RSEM was used to quantify gene abundances. Differential expression analysis was performed using the DESeq2 with *Q*-value ≤ 0.05, DEGs with | log2FC| > 1 and *Q*-value ≤ 0.05 were considered to be significantly differential expression genes (DEGs). Clusters of orthologous groups (COG) functional annotation of DEGs was obtained from eggNOG database. Gene Ontology (GO) functional enrichment analysis of DEGs were carried out by Goatools. For RT-qPCR, the total RNA of Si or CK samples were extracted with TRNzol Universal Reagent (DP424, TIANGEN, China). First-stand cDNA synthesis and qPCR were performed by using One-Step gDNA Removal and cDNA Synthesis SuperMix kit (AT311, TransGen, China) and SuperReal PreMix Plus (SYBR Green) kit (FP205, TIANGEN, China), respectively. The expression of *AtUBQ5* was used as an internal control (Nizam et al., 2019). All the primers are listed in Supplementary Table 4.

## Results

### The Growth Promotion Effect of *Serendipita indica* on Arabidopsis Are Enhanced by High Ambient Temperature

**FIGURE 1:**
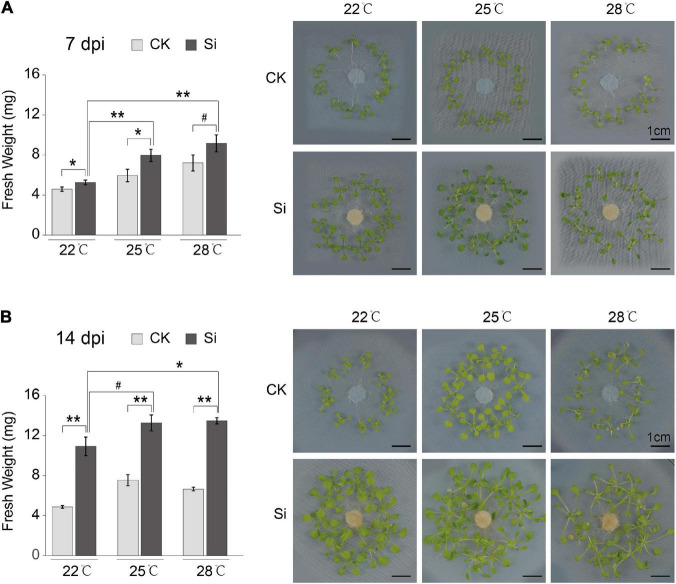
High ambient temperature strengthened the growth promotion effect of *S. indica* on Arabidopsis. *Arabidopsis thaliana* col-0 WT plants were cocultured with the endophytic fungus *S. indica* at different temperatures. *S. indica* significantly promoted the growth of Arabidopsis seedlings at 22, 25, and 28°C. The photos showed the control (CK) and *S. indica* cocultivated (Si) seedlings at the 7 days post inoculation (dpi) **(A)** and 14 dpi **(B)**. Fresh weights of seedling aboveground parts at 25 and 28°C were heavier than that at 22°C, which suggested that the growth promotion effect of *S. indica* was enhanced by elevated ambient temperature. Error bars indicate SE (*n* ≥ 3). Significance analysis of differences between CK and Si samples were performed by *t*-test (**P < 0.01; *P < 0.05; ^#^*P* < 0.1).

*Serendipita indica* colonization in roots has a significant growth promotion effect on Arabidopsis aboveground parts, but it is not known whether it is affected by high ambient temperature. We analyzed the growth promotion effect of *S. indica* on Arabidopsis plants at normal temperature (22°C) and high ambient temperatures (25 and 28°C). The fresh weights of aboveground parts of Arabidopsis inoculated with *S. indica* (Si) were significantly higher than that of control (CK) at different temperatures (Figure 1). Meanwhile, the fresh weight of the symbiotic plants grown under high ambient temperatures (25 and 28°C) were significantly higher than that under normal growth temperature (22°C) at 7 or 14 dpi (Figure 1). High ambient temperatures have significantly increased the fresh weight of the aboveground parts of Arabidopsis at 7 dpi (Figure 1A). Meanwhile, the fungal colonization rates at 7 dpi have no remarkable differences under different temperatures (Supplementary Figure 1A). In short, these results indicated that high ambient temperature enhanced the growth promotion effect of *S. indica* fungi on the aboveground parts of the host plant Arabidopsis.

### Systemic Response of Arabidopsis to *Serendipita indica* Colonization Under High Ambient Temperature

**FIGURE 2:**
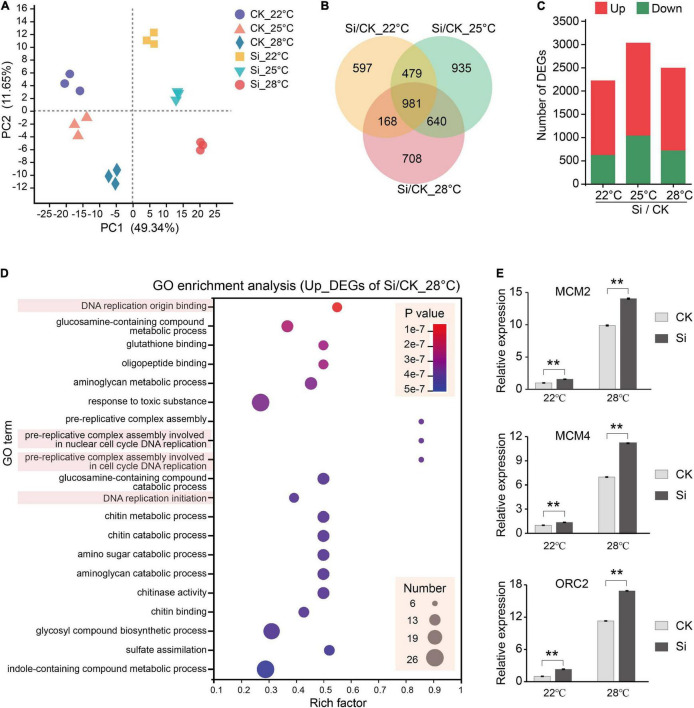
Transcriptome analysis of the symbiotic response of Arabidopsis at different temperatures. Arabidopsis seedlings incubated with *S. indica* were grown for 7 days at 22, 25, and 28°C, and the seedling aerial parts were harvested for subsequent RNA-seq and RT-qPCR analysis. **(A)** Principle component analysis (PCA) of Arabidopsis transcriptome samples. **(B)** Venn diagrams showing the number of common and specific differentially expressed genes (DEGs) between *S. indica*-incubated (Si) and control (CK) samples at 22, 25, and 28°C. **(C)** The number of DEGs upregulated or downregulated during *S. indica* colonization at different temperatures. **(D)** Gene Ontology (GO) enrichment analysis of the upregulated DEGs at 28°C, which showed that DNA replication-related genes were highly enriched. GO enrichment analysis of other DEGs is shown in Supplementary Figure 2. **(E)** The expression of the DNA replication-related genes MCM2, MCM4, and ORC2 was detected by RT-qPCR. Error bars indicate SE (*n* = 4). Significance analysis of differences were performed by *t*-test (**P < 0.01).

**FIGURE 3:**
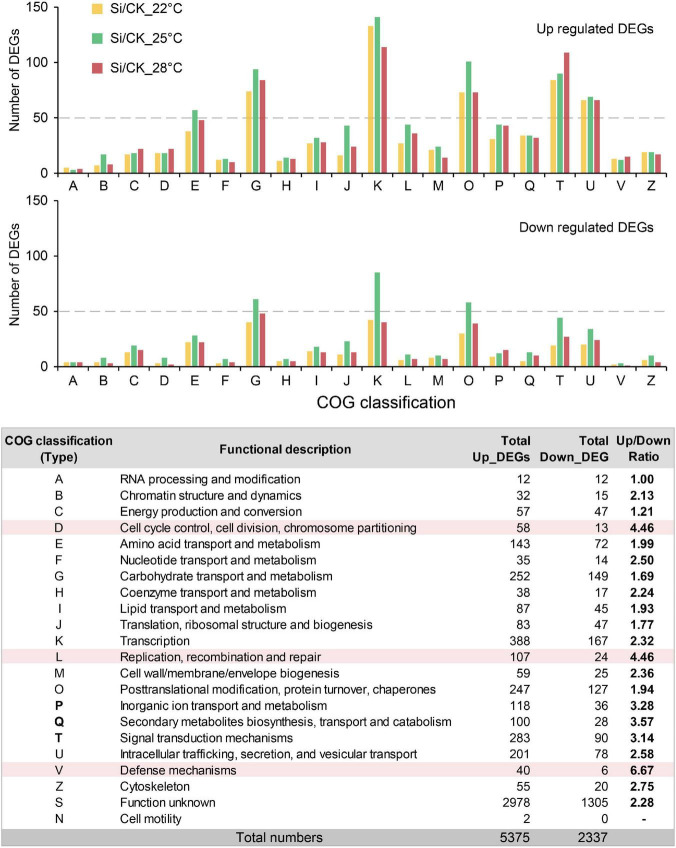
Clusters of orthologous groups (COG) analysis of DEGs during *S. indica* colonization at different temperatures. COG of proteins analysis showed that there were more upregulated DEGs than downregulated DEGs in nearly each COG classification. The ratio of upregulated DEGs to downregulated DEGs was highest in the D, L, and V types, which were related to the cell cycle and division, DNA replication and defense response. The number of DEGs in the COG classification at each temperature is shown in Supplementary Table 2.

*Serendipita indica* colonization in Arabidopsis roots has several typical phases: the early biotrophic phase at about 3 dpi, the late biotrophic phase at about 7 dpi and the late saprotrophic phase at about 14 dpi (Zuccaro et al., 2011; Lahrmann et al., 2013, 2015). The intracellular colonization of *S. indica* were well established at 7 dpi (Lahrmann et al., 2015). To understand the mechanism underlying the *S. indica*-induced plant growth-promoting effect, we attempt to compare transcriptional changes in the aboveground parts of the host plant Arabidopsis. In our experimental system, *S. indica* also well colonized Arabidopsis roots and had significant growth promoting effects at 7 dpi (Figure 1A and Supplementary Figure 1A). Thus, we harvested the aboveground parts of Arabidopsis seedlings at 7 dpi under different temperatures for subsequent transcriptome analysis (Figure 2A). We compared the DEGs between *S. indica* incubated- and sterile cultured-samples at 22, 25, and 28°C separately, and found that a large part of the DEGs (that is, 981) were commonly regulated at different temperatures (Figures 2A,B). Under each temperature condition, the number of upregulated DEGs (1,601 approximately 72% at 22°C, 1,997 approximately 65.8% at 25°C, and 1,774 approximately 71% at 28°C) was greater than that of downregulated DEGs (624 approximately 28% at 22°C, 1,038 approximately 34.2% at 25°C, and 723 approximately 29% at 28°C) (Figure 2C and Supplementary Table 1). Then, we analyzed the upregulated and downregulated DEGs. GO analysis of upregulated DEGs showed that GO terms of “DNA replication origin binding,” “pre-replicative complex assembly involved in cell cycle DNA replication,” and “DNA replication initiation” were enriched (Figure 2D and Supplementary Figure 2), and upregulated DEGs in these DNA replication-related GO terms were closely related to promoting the cell cycle and growth. For the downregulated DEGs, photosynthesis-related GO terms, such as “photosynthesis, light harvesting in photosystem I,” etc. (Supplementary Figure 2), were enriched. Meanwhile, COG analysis also showed that there were many more upregulated DEGs than downregulated DEGs in the COG classifications of “Cell cycle control, cell division, chromosome partitioning” and “Replication, recombination and repair” (Figure 3 and Supplementary Table 2). In the COG classification of “Replication, recombination and repair,” the number of upregulated DEGs at high ambient temperature (44 at 25°C and 36 at 28°C) was greater than that at normal temperature (27 at 22°C) (Supplementary Table 2). Meanwhile, RT-qPCR analysis further confirmed that, compared with *S. indica* colonized seedlings grown under normal temperature (22°C), the gene expression of key DNA replication proteins, including MCM2, MCM4, and ORC2 (Collinge et al., 2004; Diaz-Trivino et al., 2005; Shultz et al., 2009), was more increased at high ambient temperature (28°C) (Figure 2E). Collectively, these data suggested that DNA replication-related pathways in the aboveground parts of host plants are activated by *S. indica* colonization, especially under high ambient temperature. These data supported the results in Figure 1 that high ambient temperature enhanced the growth-promoting effect of *S. indica* on the aboveground parts of Arabidopsis.

### High Ambient Temperatures Repressed the Jasmonic Acid and Ethylene Signaling Pathways During the Plant Systemic Response

**FIGURE 4:**
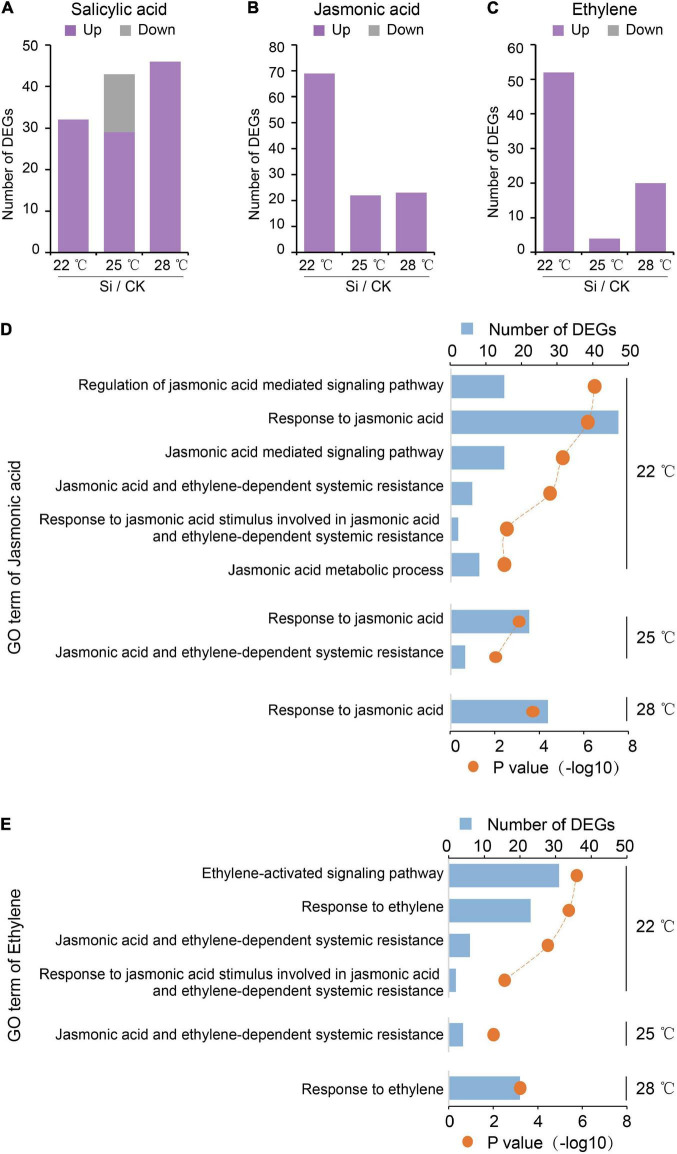
High ambient temperature repressed plant JA and ET pathways during the systemic response. High temperature affected the number of DEGs between *S. indica*-incubated (Si) and control (CK) samples. **(A)** The number of DEGs of the salicylic acid (SA) pathway in Arabidopsis aboveground parts. **(B,C)** High ambient temperatures decreased the number of DEGs in the jasmonic acid (JA) and ethylene (ET) pathways. These DEGs were all upregulated DEGs. These results suggested that Arabidopsis JA and ET pathways induced by *S. indica* colonization are downregulated by elevated temperature. **(D,E)** GO analysis to the DEGs of JA and ET pathways.

In addition to regulating plant growth, high ambient temperature also strongly suppresses defense responses (Alcazar and Parker, 2011). Plant hormones, such as SA, JA, and ET, play important roles in the induced systemic defense responses of plants (Pieterse et al., 2014). JA and ET also involved in the Arabidopsis-*S. indica* interaction (Stein et al., 2008; Camehl et al., 2010; Lahrmann et al., 2015). Here, our transcriptome analysis showed that DEGs involved in the JA and ET pathways were all upregulated DEGs. High ambient temperatures greatly decreased the number of upregulated DEGs in the JA and/or ET signaling pathways but not in SA-related GO terms (Figure 4). These results suggested that the Arabidopsis JA and/or ET signaling pathways were activated and involved in the *S. indica*-induced systemic response, and were repressed by high ambient temperature.

### pif4 Mutant Enhanced the Growth Promotion Efficiency of *Serendipita indica* on Arabidopsis at High Ambient Temperatures

**FIGURE 5:**
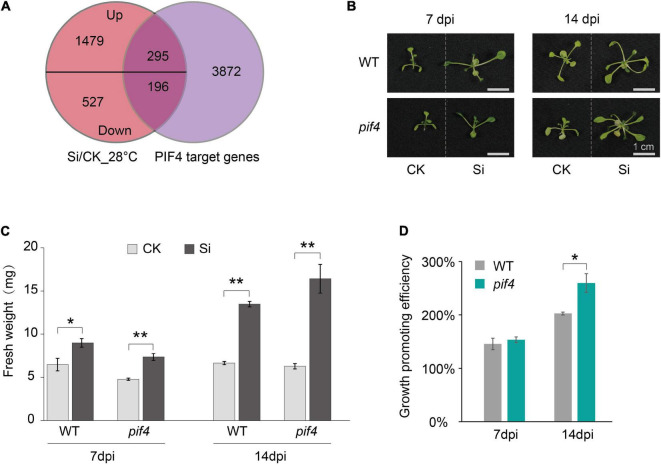
PIF4 regulated the growth promotion efficiency of *S. indica* in Arabidopsis under high ambient temperature. **(A)** Venn diagram showing that PIF4 target genes overlapped with many upregulated or downregulated DEGs during the systemic response of Arabidopsis aboveground parts to *S. indica* at high ambient temperature (28°C). **(B)** *S. indica*-incubated seedlings (Si) at 7 or 14 dpi and their control (CK) seedlings. **(C)** The fresh weight of wild-type (WT) and *pif4* plants at 7 and 14 dpi when cocultured with *S. indica* at 28°C. **(D)** The growth promotion efficiency of *S. indica* on *pif4* (*pif4-101*, a *pif4* loss-of-function mutant) plants was higher than that on WT plants at 28°C. Growth promoting efficiency was obtained by dividing the fresh weight of Si samples by that of CK samples. Error bars indicate SE (*n* ≥ 3). Significance analysis of differences were performed by *t*-test (**P < 0.01; *P < 0.05).

Arabidopsis JA and/or ET signaling pathways are also regulated by PIF4 (Yamashino et al., 2013; Zhang et al., 2018; Xiang et al., 2020). More importantly, PIF4 is the central hub transcription factor that regulates the aboveground tissue response to high ambient temperature in Arabidopsis (Lee et al., 2021; Zhang et al., 2021). Therefore, we wondered whether PIF4 is involved in the systemic response of Arabidopsis aboveground tissue to *S. indica* colonization under high ambient temperatures. Previous studies have reported 4,362 PIF4-binding target genes (Oh et al., 2012), so we first compared PIF4 targets with those DEGs during *S. indica* colonization. The Venn diagram shows significant overlap between PIF4-binding target genes and DEGs at high ambient temperature (28°C) (Figure 5A and Supplementary Table 3). Then, we incubated *S. indica* with a loss-of-function *pif4* mutant (*pif4-101*) at 28°C and analyzed the fresh weight of their aboveground tissues. Interestingly, the growth promotion effect of *S. indica* on the *pif4* mutant was significantly stronger than that on WT plants at 14 dpi under 28°C (Figures 5B–D). Meanwhile, the fungal colonization rate between *pif4* mutant and WT plants have no significant difference at 14 dpi under 28°C (Supplementary Figure 1B). These data suggest that *PIF4* was involved in the response of Arabidopsis aboveground tissues to high ambient temperature during beneficial endophytic fungal colonization.

## Discussion

Soil fungi in nature establish beneficial symbiotic relationships with most vascular plants (Brundrett and Tedersoo, 2018; Genre et al., 2020). Beneficial endophytic fungi in roots promote the induced systemic resistance of plant aboveground parts to pathogenic microorganisms, herbivorous insects and abiotic stresses and promote the growth of plant aboveground parts (Franken, 2012; Cameron et al., 2013; Pieterse et al., 2014). Over the past decades, significant progress has been achieved in understanding the molecular mechanism of induced systemic resistance in plants, especially the systemic response induced by pathogenic microorganisms (Jung et al., 2012; Walters et al., 2013; Pieterse et al., 2014; Hilleary and Gilroy, 2018). However, the mechanisms by which it promotes plant growth are not well studied. *S. indica* colonization in roots has a significant growth promotion effect on many host plants (Qiang et al., 2012). Here, we examine the transcriptome response of aboveground parts of Arabidopsis plants to *S. indica* colonization under normal or high ambient temperatures. Compared with sterile seedlings, DEGs related to DNA replication were significantly enriched and upregulated in plants cocultured with *S. indica* (Figures 2, 3). These data are consistent with the result that higher ambient temperatures can enhance the growth-promoting effect of *S. indica* on the aboveground parts of Arabidopsis (Figure 1). By the way, photosynthesis-related GO terms were enriched in the downregulated DEGs (Supplementary Figure 2). Photosynthetic efficiency is vital for promoting plant biomass, and *S. indica* symbiosis promotes the photosynthesis of host plants (Li et al., 2021). Thus, the downregulation of photosynthesis-related genes may be feedback regulation from the increased photosynthesis efficiency of host plants.

Plant hormones (SA, JA, ET, etc.) play major roles in plant systemic responses, including defense and growth-promoting effects (Pieterse et al., 2014; Xu et al., 2018; Dai et al., 2019). *S. indica* colonization increased JA accumulation and decreased SA levels in Arabidopsis roots (Lahrmann et al., 2015). The systemic resistance response induced by *S. indica* colonization was independent of SA signaling but required an operative JA defense pathway (Stein et al., 2008). Overexpression of ETHYLENE RESPONSE FACTOR1 (ERF1), which directly activates many ET-inducible defense genes, strongly reduces *S. indica* colonization in roots and abolishes growth promotion in the aboveground part of Arabidopsis (Camehl et al., 2010). Here, we found that the number of DEGs related to the JA and ET pathways under high ambient temperatures was significantly decreased compared to that under normal temperature (Figure 4), suggesting that JA and/or ET signaling pathways were repressed under high ambient temperature. Activation of JA and ET signals in leaves inhibited plant growth by inhibiting cell division (Huang et al., 2017; Dubois et al., 2018). Thus, the repression of JA and/or ET signaling pathways (Figure 4) and the upregulation of DNA replication related genes (Figure 2) should all contribute to the higher growth promotion effects under high ambient temperature. Furtherly, the growth promotion and defense inhibition are antagonistically linked in plants (Huot et al., 2014; Guo et al., 2018). High ambient temperature promotes growth, whereas it suppresses defense responses in plants (Alcazar and Parker, 2011). Thus, the increased growth promotion efficiency under high ambient temperatures (Figures 1, 4) may be associated with inhibition of JA and/or ET-related defense responses. The role of JA and ET pathways in plant growth-defense trade-offs deserves attention in the future study of plant systemic responses under high ambient temperatures. Meanwhile, carbon availability has been suggested to modulate the plant growth-defense trade-offs, in which the activation of JA signaling pathway depletes sucrose and starch content (Smith and Stitt, 2007; Machado et al., 2017; Guo et al., 2018). The inhibition of JA signaling pathway by high ambient temperature may inhibit carbon consumption for defense response, thus allow more carbon metabolites to be used for plant growth.

The Arabidopsis PIF4 transcription factor plays a central role in the response to high ambient temperature (Koini et al., 2009; Zhang et al., 2021). PIF4 is also involved in the regulation of the JA and/or ET signaling pathways (Yamashino et al., 2013; Zhang et al., 2018; Xiang et al., 2020). Loss-of-function mutations of pif4 inhibit Arabidopsis petiole elongation and other phenotypes during thermosensory growth (Koini et al., 2009; Zhang et al., 2021). However, it should be noted that seedlings overexpressing PIF4 grew very thin and had significantly lower biomass than WT plants, especially at high ambient temperature (Kumar et al., 2012). Here, we found that the *pif4* mutant promoted the increase in aboveground biomass induced by *S. indica* colonization under high ambient temperature (Figure 5). DEGs that regulate the systemic response to *S. indica* colonization and the target gene of PIF4 exhibited considerable overlap (Figure 5). In short, PIF4 is involved in the Arabidopsis systemic response and regulates the growth promotion effect of *S. indica* on aboveground parts of plants under high ambient temperature. The detailed mechanism of PIF4 regulating the growth promotion effect of *S. indica* on plants needs to be further studied, which may be through JA and/or ET signaling pathway.

A recent transcriptome study showed that only a small portion of systemic response DEGs overlapped with local response DEGs during abiotic stress in plants (Zandalinas et al., 2020). COG analysis to transcriptome data showed that most DEGs (2,978 upregulated DEGs and 1,305 downregulated DEGs) under different temperatures belonged to Function unknown (S) classification (Figure 3). Future studies on these DEGs with unknown functions will reveal the detailed molecular mechanisms by which beneficial endophytic symbiosis regulates the systemic response of aboveground tissues under high and/or normal ambient temperature conditions.

## Data Availability Statement

The data presented in the study are deposited in the Gene Expression Omnibus (GEO) under accession number GSE197325.

## Author Contributions

JJH and XJC designed the experiments, analyzed the data, and wrote the manuscript. XJC, YQY, XMZ, and XX performed the experiments. All authors contributed to the article and approved the submitted version.

## Conflict of Interest

The authors declare that the research was conducted in the absence of any commercial or financial relationships that could be construed as a potential conflict of interest.

## Publisher’s Note

All claims expressed in this article are solely those of the authors and do not necessarily represent those of their affiliated organizations, or those of the publisher, the editors and the reviewers. Any product that may be evaluated in this article, or claim that may be made by its manufacturer, is not guaranteed or endorsed by the publisher.

## Footnotes

1
www.majorbio.com
