# Supplementary material for: High Ambient Temperature Regulated the Plant Systemic Response to the Beneficial Endophytic Fungus Serendipita indica
Source: Front Plant Sci. 2022 Mar 16;13:844572. doi: 10.3389/fpls.2022.844572 (PMC8966885; doi:10.3389/fpls.2022.844572)
Supplement: Supplementary file 4 [file Data_Sheet_1.pdf]

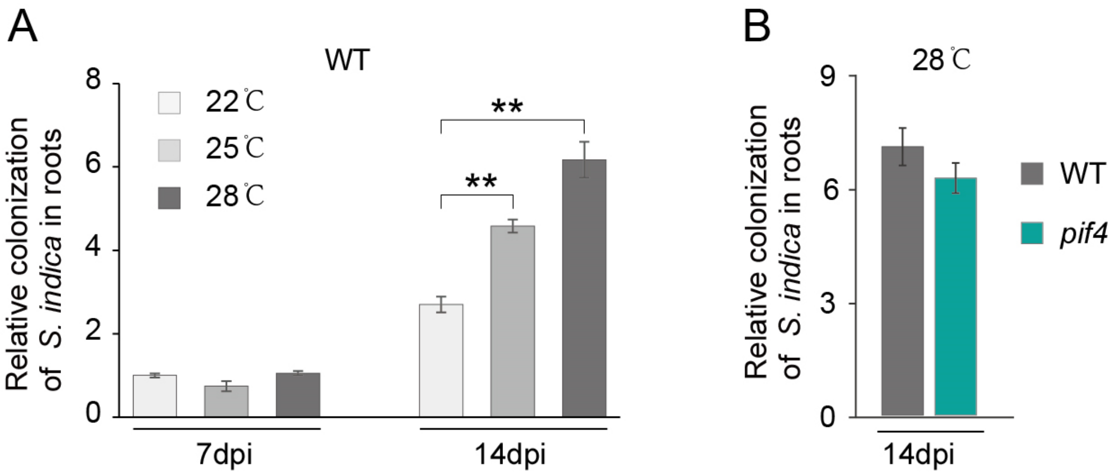

Figure S1. The relative colonization level of *S. indica* in Arabidopsis roots

(A) The relative colonization level of *S. indica* in wild type (WT) Arabidopsis roots at 7 days post incubation (dpi) or 14dpi under 22°C, 25°C and 28°C. **(B)** The relative colonization level of *S. indica* in the roots of WT and *pif4* mutant (*pif4-101*) plants at 14 dpi under 28°C. The relative colonization level of *S. indica* were detected by RT-qPCR analysis of *SiTef1* normalized to the expression of *AtUBQ5* (Nizam et al., 2019). Error bars indicate SE (n≥3). Significance analysis of differences were performed by t-test (***, P <0.01*).

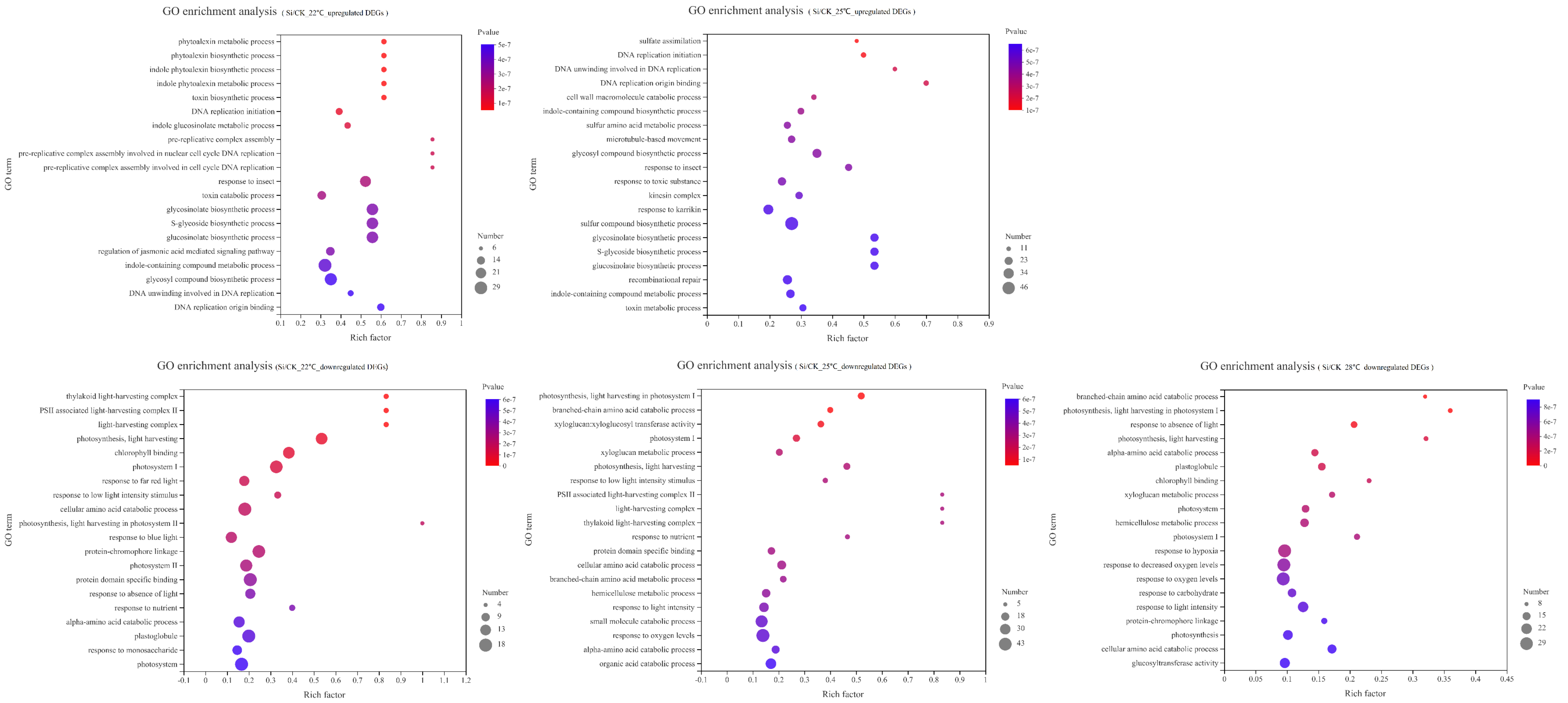

Figure S2. GO enrichment analysis of the upregulated and downregulated DEGs

Gene ontology (GO) enrichment analysis were performed for the upregulated differentially expressed genes (DEGs) between mycorrhizal symbiotic and nonsymbiotic plants at 22°C and 25°C, and the downregulated DEGs at 22°C, 25°C and 28°C.

Table S4: Primers used in this study

Name	Sequence（5’-3’）	Remarks
AtUBQ5-F257	CCAAGCCGAAGAAGATCAAG	qPCR for AT3G62250 (*AtUBQ5*)
AtUBQ5-R361	ACTCCTTCCTCAAACGCTGA	
MCM2-F2423	GGCAATATGTCACAGAAGAGGAT	qPCR for AT1G44900 (*MCM2*)
MCM2-R2583	GAGAAGAACGAGCAGCAATGA	
MCM4-F1830	CCTTCCTCCAACCTTGCTATC	qPCR for AT2G16440 (*MCM4*)
MCM4-R1907	CTTCGGTCAGTCTGCTCATC	
ORC2-F568	CTAAGAGATCCCGAATCACAACAA	qPCR for AT2G37560 (*ORC2*)
ORC2-R642	AATAGAGGCAACCAAGCGTATG	
SiTef1-qF3089	GCAAGTTCTCCGAGCTCATC	RT-qPCR for AJ249911 (*SiTef1*)
SiTef1-qR3245	CCAAGTGGTGGGTACTCGTT	
